# Seasonal Trade-Offs and Synergies Between Food Resources and Solar Radiation Shape Altitudinal Ranging in Black-and-White Snub-Nosed Monkeys

**DOI:** 10.3390/ani16152280

**Published:** 2026-07-23

**Authors:** Hao-Han Wang, Yan-Peng Li, Zhi-Pang Huang, Liang-Wei Cui, Cyril C. Grueter, Na Li, Wen Xiao

**Affiliations:** 1 Institute of Eastern-Himalaya Biodiversity Research, Dali University, Dali 671003, China; wanghh@eastern-himalaya.cn (H.-H.W.); liyp@eastern-himalaya.cn (Y.-P.L.); xiaow@eastern-himalaya.cn (W.X.); 2 Research Center of Natural History and Culture, Qujing Normal University, Qujing 655011, China; 3 Key Laboratory for Forestry Disaster Warning and Control in Yunnan, Southwest Forestry University, Kunming 650224, China; cuilw@eastern-himalaya.cn; 4 Yunling Black-and-White Snub-Nosed Monkey Observation and Research Station of Yunnan Province, Dali 671003, China; 5 Institute of Human Sciences, School of Anthropology and Museum Ethnography, University of Oxford, Oxford 02138, UK; cyril.grueter@anthro.ox.ac.uk; 6 International Centre of Biodiversity and Primate Conservation, Dali 671003, China; 7 Department of Anatomy, Physiology and Human Biology, School of Human Sciences, The University of Western Australia, Perth, WA 6001, Australia; 8 Centre for Evolutionary Biology, School of Biological Sciences, The University of Western Australia, Perth, WA 6001, Australia; 9 Centre of Excellence in Biodiversity and Natural Resource Management, University of Rwanda, Kigali P.O. Box 4285, Rwanda; 10 Collaborative Innovation Center for Biodiversity and Conservation in the Three Parallel Rivers Region of China, Dali 671003, China; 11 The Provincial Innovation Team of Biodiversity Conservation and Utility of the Three Parallel Rivers Region from Dali University, Dali 671003, China; 12 Center for Interdisciplinary Sciences, Dali University, Dali 671003, China

**Keywords:** high-altitude adaptation, altitudinal ranging, solar radiation, food availability, behavioral trade-off, ecological plasticity, survival strategy

## Abstract

High-altitude animals rely on vertical spatial adaptation to cope with harsh environments, yet the underlying drivers of their seasonal altitudinal distribution remain poorly understood. This study investigated how solar radiation, seasonal high-quality food resources and stable foods shape the altitudinal distribution of the highest-altitude primate *Rhinopithecus bieti*, to uncover its survival strategies. Through systematic environmental data collection and three-year field tracking of a wild group in the Lasha Mountains, northwest Yunnan, China, we identified consistent seasonal patterns: spring in low-altitude habitats, summer at highest elevations, and intermediate ranges during autumn/winter. Statistical modeling revealed seasonally shifting environmental dependencies. Summer showed no resource limitations, while autumn introduced solar exposure and mature leaves as positive synergistic factors (50% model explanatory power). Winter exhibited peak environmental pressure (80%), with solar and buds becoming critical, though revealing a trade-off between foraging and thermoregulation. Spring demonstrated the strongest environmental coupling (86%), where solar and high-quality food showed significant positive synergy, enabling physical recovery. The species displays remarkable ecological plasticity, with solar being the core cold-season driver. The winter-to-spring transition from resource trade-off to synergy reflects dynamic behavioral adaptations to seasonal climate and nutritional bottlenecks, offering new insights into primate survival strategies in extreme environments.

## Simple Summary

Animals living at high elevations must adjust their movements to cope with seasonal changes in temperature and food availability, yet the factors driving these movements remain poorly understood. We studied the black-and-white snub-nosed monkey (*Rhinopithecus bieti*), the world’s highest-altitude nonhuman primate, to determine how solar radiation and seasonal food resources influence its altitudinal ranging. Based on three years of field observations in Yunnan, China, we found a consistent annual pattern: monkeys occupied the lowest elevations in spring, the highest elevations in summer, and intermediate elevations in autumn and winter. The environmental factors shaping these movements changed seasonally. During summer, food and thermal conditions imposed few constraints, whereas autumn habitat use was associated with both solar radiation and mature leaves. In winter, monkeys balanced access to food with warmer, sunnier habitats, while in spring they benefited from the combined availability of solar radiation and nutrient-rich foods. These findings demonstrate the remarkable behavioral flexibility of this high-altitude primate and improve our understanding of how mountain animals adapt to seasonal environmental change, providing valuable information for their conservation.

## 1. Introduction

High-mountain environments are characterized by harsh climatic conditions, including low temperatures, hypoxia, and seasonal fluctuations in food availability, while simultaneously providing diverse habitat opportunities across elevational gradients. To survive in these challenging environments, animals have evolved a range of adaptations, including morphological modifications [1], genetic adaptations [2], and physiological and behavioral adjustments [3]. Among these, seasonal altitudinal ranging is a particularly important behavioral strategy that enables animals to track favorable environmental conditions and resources throughout the annual cycle [4,5]. This form of movement is widespread among mountain-dwelling species and represents a key mechanism for coping with seasonal environmental variation [6]. Similar to latitudinal migration, altitudinal migration allows animals to exploit spatiotemporal variation in habitat quality; however, it generally incurs lower energetic costs because of shorter travel distances and requires fewer specialized morphological and physiological adaptations [7]. Understanding how animals make movement decisions in response to environmental heterogeneity remains a central challenge in ecology and has been identified as one of the fundamental questions in the study of animal migration and movement ecology [8].

Food availability and climatic conditions are widely recognized as the primary extrinsic drivers of altitudinal migration in animals [7]. In addition to these core factors, other environmental variables, including nest predation risk and anthropogenic disturbances, can also influence elevational movement patterns [9]. Despite growing interest in altitudinal migration, research remains taxonomically biased. Birds have received the greatest attention, whereas studies of mammals are comparatively limited [7]. Among mammals, most investigations have focused on ungulates, for which food quantity, forage quality, and snow cover have been identified as the principal determinants of seasonal altitudinal movements [7,10,11]. However, elevational migration can be driven by different mechanisms in other taxa [12]. For example, the seasonal upward migration of giant pandas (*Ailuropoda melanoleuca*) is associated with both increasing temperatures at lower elevations and shifts in the distribution of preferred bamboo resources [13]. These findings highlight the interactive roles of climate and resource dynamics in shaping elevational movement strategies. More broadly, such patterns are consistent with Rapoport’s altitudinal rule, which predicts that high-elevation species generally occupy broader elevational ranges than low-elevation species because adaptation to greater climatic variability confers wider environmental tolerance [14].

Despite the numerous factors proposed to influence altitudinal migration, empirical evidence remains limited, and the extent to which these drivers can be generalized across taxa is still poorly understood [15,16]. One major challenge is the difficulty of obtaining detailed, long-term records of animal movements across heterogeneous mountain landscapes [17]. Fine-scale information on population movements is essential for understanding how animals adjust their behavior in response to seasonal environmental variation, particularly for species with large home ranges and complex movement patterns.

The black-and-white snub-nosed monkey (*Rhinopithecus bieti*) is the highest-altitude dwelling nonhuman primate, inhabiting cold subalpine forests across an elevational range of approximately 2600–4500 m [18]. Because of its dependence on highly seasonal environments, this species provides a valuable model for investigating the ecological drivers of altitudinal ranging behavior. However, our understanding of the mechanisms underlying its elevational movements remains limited. First, although several studies have described the seasonal altitudinal ranging patterns of *R. bieti*, the consistency of these patterns across years and populations remains insufficiently understood. Although several studies have examined habitat use and elevation preferences, few have conducted continuous year-round observations (but see [19]), and none has monitored the same population over multiple consecutive years. As a result, contrasting patterns have been reported, even within the same study area [19,20,21]. Most studies suggest that *R. bieti* occupies lower elevations in spring, ascends to higher elevations in summer, and uses intermediate elevations during autumn and winter [19,21,22,23]. In contrast, other studies have reported increased use of higher elevations during winter [18,20]. Second, although several hypotheses have been proposed to explain these patterns, the relative importance of different environmental drivers remains unresolved. Previous studies have highlighted either food resources and plant phenology [19,21,24] or solar radiation [18] as important determinants of altitudinal habitat use. However, no study has explicitly evaluated how food resources and solar radiation jointly influence seasonal altitudinal ranging patterns in *R. bieti*. Consequently, the mechanisms through which food resources and thermal conditions jointly shape altitudinal ranging behavior in *R. bieti* remain poorly understood.

Long-term observations by our research team indicate that lichens constitute the primary food resource for *R. bieti*, accounting for approximately 80% of annual feeding records and up to 97% during winter [25]. In addition to this stable fallback resource, the monkeys exploit seasonally available high-quality foods, including buds and young leaves in spring, flowers and leaves in summer, and fruits in autumn [25]. The availability of these foods varies markedly across elevations and seasons, whereas lichens remain abundant and broadly distributed throughout the year. Given the strong seasonal variation in food quality and availability, together with the energetic challenges imposed by low temperatures at high elevations, both food resources and solar radiation are expected to play important roles in determining habitat selection and altitudinal ranging patterns.

Therefore, the primary objectives of this study were to (1) characterize the annual altitudinal ranging patterns of a wild *R. bieti* group based on three consecutive years of field observations, and (2) evaluate the relative and interactive effects of food resources and solar radiation on seasonal habitat use. By integrating resource availability and thermal environmental conditions, this study provides new insights into the behavioral strategies that enable high-altitude primates to maintain energy balance and cope with seasonal environmental constraints.

## 2. Materials and Methods

### 2.1. Study Area

Field work was conducted on one large free-ranging group of the *R. bieti* in Lasha Mountains (26°20′ N, 99°15′ E), Lanping County, Yunling Nature Reserve in the northwest of Yunnan Province. The group’s home range is well defined and stable across years, and its size has remained largely consistent (~120 individuals) throughout the observation periods. Therefore, group identity does not serve as a confounding variable in our analyses of elevational distribution. The study group occupied an area of approximately 1400 ha, ranging from 2800 m near the valley bottom to 3855 m above sea level near the mountain summits. Mean annual precipitation is approximately 970 mm, and mean annual temperature is 11.7 °C. Both temperature and precipitation exhibit pronounced seasonal variation [26]. Vegetation changes markedly along the elevational gradient. Lower elevations are dominated by deciduous broadleaved forests consisting primarily of *Quercus* spp., *Sorbus* spp., and *Acanthopanax evodiaefolius*. At intermediate elevations, these forests transition into mixed deciduous broadleaf–conifer forests dominated by *Abies georgei*, *Tsuga dumosa*, and *Picea likiangensis*. Higher elevations are characterized by dark conifer forests dominated by *Abies georgei*, *Abies fabri*, and *Tsuga dumosa*. The understory vegetation is composed mainly of *Rhododendron* spp. and bamboo species (*Fargesia strigosa*, *F. edulis*, and *F. solida*).

### 2.2. Location Recording of Rhinopithecus bieti

**Figure 1:**
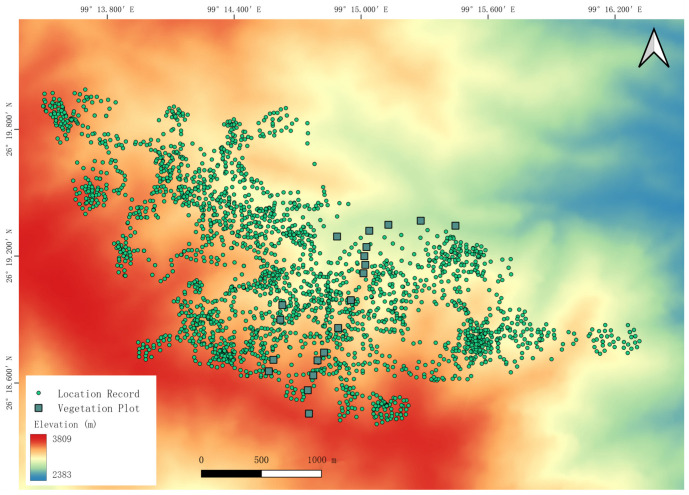
Spatial distribution of *Rhinopithecus bieti* location records and vegetation plots across the elevational gradient of the study area.

A total of 6451 location records, including altitude and geographic coordinates of the group’s center, were collected over 399 observation days spanning three annual cycles (Figure 1). Data were collected by Zhi-Pang Huang from May 2008 to September 2009, Shuang-Jin Wang from August 2010 to November 2012, and Yun Wang from September 2014 to August 2015. Field observations were conducted by one or two observers per month. Observers maintained distances of approximately 80–800 m from the monkey group. Group center locations were determined by visually estimating group spread and recording the midpoint using a Garmin eTrex Summit GPS receiver (Garmin, Taiwan, China). At each location, habitat type and the monkeys’ predominant activity were recorded. After the monkeys moved away from a recorded location, observers approached or walked to the actual site to verify and correct the coordinate using a handheld GPS receiver in conjunction with printed topographic maps and high-resolution satellite imagery. This post hoc ground-truthing procedure minimized positional errors arising from long-distance visual estimation, resulting in a final positional accuracy generally within 15 m for all occurrence records. In addition, movement trajectories between consecutive locations were documented by following the group throughout the observation period.

### 2.3. Phenology and Food Availability

Food species were defined as plant species contributing more than 1% of feeding records and were identified based on Huang et al. [26]. Nine tree species met this criterion: *Betula alnoides*, *Acanthopanax gracilistylus*, *Acer oliverianum*, *Sorbus glomerulata*, *Sorbus megalocarpa*, *Ilex polyneura*, *Prunus pilosuscula*, *Populus adenopoda*, and *Cornus macrophylla*. Two fruticose lichen species, *Bryoria* sp. and *Usnea longissima*, were also included because of their importance in the monkeys’ diet. The abundance and stem density of these food tree species across the elevational gradient were assessed following the methods of Huang et al. [26]. A total of 140 vegetation plots, representing approximately 0.2% of the group’s home range, were surveyed.

To quantify seasonal variation in food availability, phenological monitoring was conducted monthly from June 2008 to September 2009. Twenty vegetation plots were established along two elevational transects spanning 2850–3650 m a.s.l. Plots (10 × 10 m) were located at approximately 50 m elevational intervals where suitable forest habitat occurred (Figure 1). For each food tree species, one representative individual was selected within or nearest to each plot, yielding up to 20 monitored trees per species. During the second week of each month, the percentage cover of buds, young leaves, flowers, and fruits within the crown of each monitored tree was visually estimated. Lichen availability was assessed simultaneously. For each plot, the coverage of *Bryoria* sp. and *Usnea longissima* was visually estimated on individuals of the 15 most abundant tree species. Mean lichen coverage across all sampled trees within a plot was used as an index of lichen availability. Although our investigation of food resources did not cover the entire period of the behavior survey, the analysis revealed that annual rainfall (691~1149 mm, SE = 125.9) and temperature (11.8~12.3 °C, SE = 0.4) were relatively stable throughout the entire behavior survey period. Therefore, we thought that the obtained data on food resources can represent the characteristics of the period from 2008 to 2015.

### 2.4. Data Analysis

To evaluate whether seasonal altitudinal ranging patterns were consistent across annual cycles, we compared the elevational distributions of *R. bieti* among years. Seasonal probability density distributions of monkey locations were plotted for each annual cycle. Differences in altitude use among seasons were assessed using one-way analysis of variance (ANOVA), followed by pairwise Wilcoxon rank-sum tests for post hoc comparisons. Because vegetation phenology in high-altitude environments does not necessarily correspond to calendar seasons, seasonal boundaries were defined using vegetation dynamics. We obtained Normalized Difference Vegetation Index (NDVI) data from the MOD13Q1 product [27] for the period 2000–2011. Based on annual NDVI trajectories in the Lasha Mountains, four phenologically distinct seasons were identified and used in subsequent analyses.

To examine the potential influence of solar radiation on seasonal habitat use, we quantified spatial variation in solar radiation across the study area and related it to the altitudinal distribution of monkey groups. Remote-sensing and topographic data were obtained from the NASA Land Processes Distributed Active Archive Center (LP DAAC; https://lpdaac.usgs.gov/). Specifically, we used (1) the MOD13Q1 Version 6 product to obtain solar geometric parameters, including solar zenith angle (SZA), view zenith angle (VZA), and relative azimuth angle (RAA), and (2) the Shuttle Radar Topography Mission (SRTM) digital elevation model (DEM) at 30 m spatial resolution to characterize terrain effects on solar radiation. To ensure consistency between datasets, the 30 m DEM was resampled to 250 m using bilinear interpolation to match the spatial resolution of the MOD13Q1 product. Elevation, slope, aspect, and terrain-shading indices were derived from the resampled DEM and used to account for topographic influences on incoming solar radiation. These variables were subsequently incorporated into seasonal analyses of habitat use and altitudinal distribution.

To quantify spatial variation in solar radiation, we applied the topographic-corrected solar radiation model developed by Allen et al. [28], which has been widely validated in mountain ecosystem studies. First, extraterrestrial solar radiation (ESR), representing the theoretical maximum solar radiation at the top of the atmosphere, was calculated for each pixel based on geographic coordinates, MOD13Q1 composite dates, and solar zenith angle (SZA). Second, terrain effects on solar radiation were corrected using slope, aspect, and topographic shading factors derived from the DEM. These corrections accounted for variation in solar incidence angle and distinguished direct and diffuse radiation components, thereby generating estimates of actual surface solar radiation for each pixel. Because winter represents the period of greatest thermal stress for *R. bieti*, winter-solstice solar radiation was used as an index of habitat thermal conditions. Surface solar radiation values corresponding to monkey occurrence records obtained during field surveys were extracted from the winter-solstice solar radiation layer and used as independent variables in subsequent Generalized Linear Models (GLMs) to evaluate the influence of solar radiation on the elevational distribution of *R. bieti*. Winter solstice surface solar radiation was chosen because it captures the period of minimal solar elevation, maximizing topographically driven variation among sites. Moreover, winter solstice radiation was strongly correlated with radiation in other seasons across our study sites (Pearson correlation test; r > 0.92, *p* < 0.01), confirming that it reliably ranks locations by relative solar exposure throughout the year.

To assess whether food resource influences the seasonal distribution pattern of *R.bieti* monkeys in Lasha Mountains, we analyzed the monkeys’ altitudinal distribution in relation to food availability during one annual cycle. Food resources were categorized as either high-quality foods or stable foods. High-quality foods include buds, young leaves, flowers, and fruit, which typically exhibit seasonal fluctuations and are rich in nutrients. Stable foods consist of lichens (*Bryoria* sp. and *Usnea longsissima*) and grown-up leaves of certain plant species such as *Prunus pilosuscula*. Lichen is a slow-growing organism with an annual increment of only up to 5 cm in our study area, making seasonal variation in its biomass and chemical composition negligible. We calculated the high quality food availability index (FAI) for each of the 16 elevational belts (i = 1 to 16, from 2850 to 3650 m a.s.l., divided into 50-m elevational intervals), considering the nine food tree species (j = 1 to 9) and four food components (c = 1 to 4: buds, tender/young leaves, flowers, and fruits), following the regulation outlined by Dasilva [29]:

FAI_i_ = Coverage_c,j,i_ × Stem Density_j,i_ × Area Percentage_i_.

where Coverage_c,j,i_ is the proportional abundance of food component c of species j within elevational belt i, StemDensity_j,i_ is the stem density of species j within elevational belt i, and Area Percentage_i_ is the proportion of the study area represented by elevational belt i.

To determine the area percentage of each elevational belt, the 1400-ha study area was divided into 20 m × 20 m quadrats (N = 350). The midpoint elevation of each quadrat was used to assign it to an elevational belt. The number of quadrats within each elevational belt (n_i_) was counted, and the area percentage of that belt was calculated as n_i_/N. Using this approach, seasonal FAI values were calculated for high-quality foods and for mature leaves within each elevational belt. The FAI for lichens was calculated as:

FAI_i_ = Lichen Coverage_i_ × Area Percentage_i_.

where Lichen Coverage_i_ represents the mean lichen coverage within elevational belt i.

By comparing food availability indices across elevational belts and seasons, we assessed the relationship between food resources and the seasonal altitudinal distribution patterns of *R. bieti* in the Lasha Mountains.

Finally, for each season, we fitted Generalized Linear Models (GLMs) to examine the effects of solar radiation and food availability on the elevational distribution of *R. bieti*. The frequency of monkey occurrence within each elevational belt was used as the response variable, while solar radiation, high-quality food availability, mature leaf availability, and lichen availability were included as predictor variables. The following data processing and modeling procedures were applied.

a. Data Standardization

Because the environmental variables differed substantially in their units and measurement scales, all predictor variables were standardized using Z-score transformation (mean = 0, standard deviation = 1) prior to model fitting. This procedure improved model convergence and enabled direct comparison of regression coefficients, allowing the relative importance of different environmental variables to be evaluated on a common scale.

b. Model Selection

We used a negative binomial regression model within the GLM framework with a log-link function. The response variable was the frequency of monkey occurrence within each elevational belt during that particular season. Predictor variables included the availability of high-quality foods, mature leaves, and lichens within each elevational belt, as well as solar radiation values associated with monkey occurrence locations within the corresponding elevational belt.

c. Interaction Modeling and Evaluation

To examine potential interactions among environmental factors while avoiding overfitting associated with a full interaction model, we adopted a modeling strategy that included main effects and selected two-way interaction terms. Model performance was evaluated using Akaike’s Information Criterion (AIC), and the model with the lowest AIC value was considered the best-supported model. Statistical significance was assessed at α = 0.05. All data processing and statistical analyses were conducted in R (Version 4.3.0, R Core Team, https://www.r-project.org/). Negative binomial regression models were fitted using the glm.nb function in the MASS package (Version 7.3.55).

## 3. Results

### 3.1. Seasonal Altitudinal Ranging Patterns

**Figure 2:**
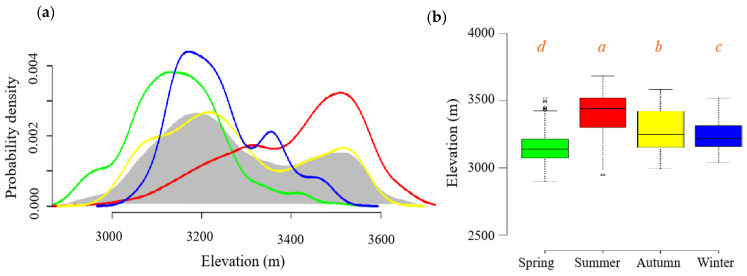
Seasonal altitudinal distribution of *R. bieti* in the Lasha Mountains. Seasonal altitudinal distribution of *R. bieti* in the Lasha Mountains. (**a**) Probability density curves show the distribution of monkey locations across the elevational gradient in each season. The gray shaded area indicates the annual elevational range of the study group’s home range. Green, red, yellow, and blue curves represent spring, summer, autumn, and winter, respectively. (**b**) Boxplots summarize seasonal elevation use. Different letters indicate significant differences among seasons based on pairwise Wilcoxon rank-sum tests (*p* < 0.05), whereas seasons sharing the same letter do not differ significantly.

**Figure 3:**
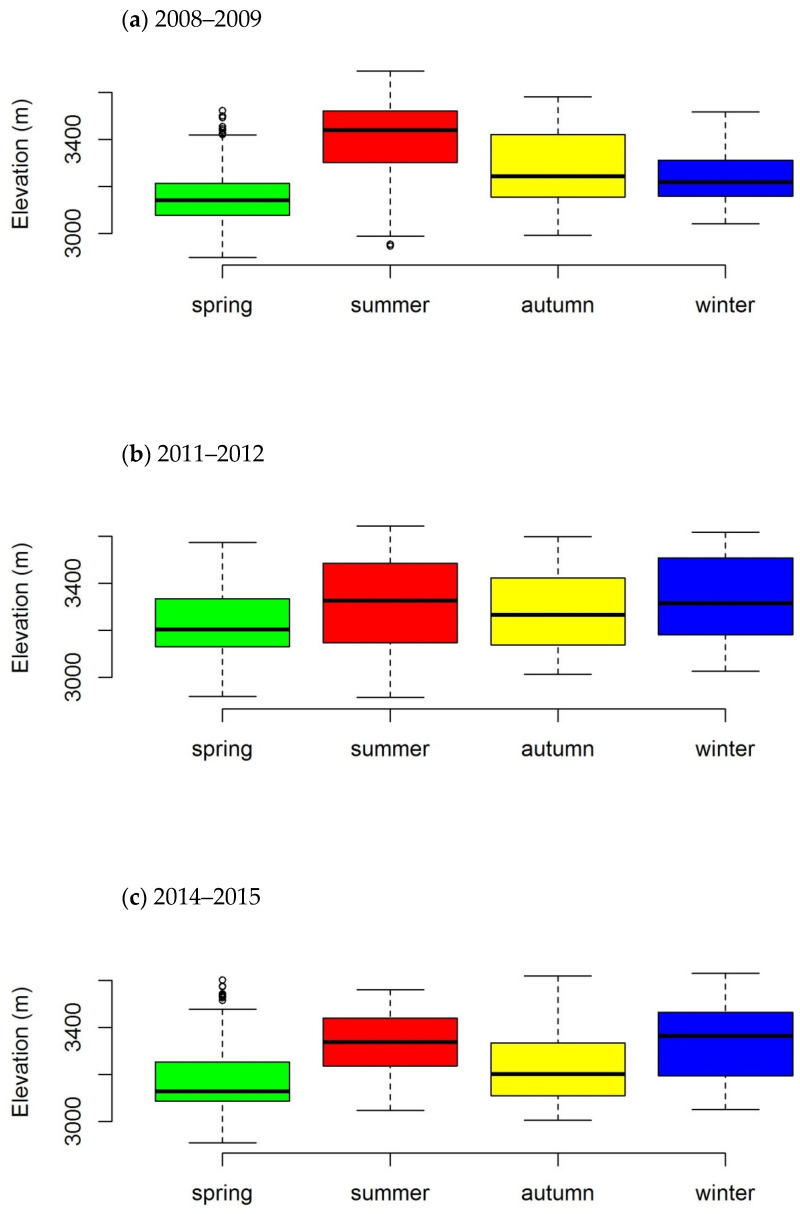
Seasonal altitudinal ranging of *R. bieti* across three annual cycles in Lasha Mountains. Boxplots show the distribution of monkey locations across the elevational gradient during spring, summer, autumn, and winter.

Based on 10-day NDVI data, the annual cycle in the Lasha Mountains was divided into four seasons: spring (March–May), summer (June–August), autumn (September–November), and winter (December–February). The altitudinal distribution of *R. bieti* varied significantly among seasons (ANOVA: F_3,3237_ = 644.4, *p* < 0.001; Figure 2). The monkeys occupied the lowest elevations during spring and the highest elevations during summer. In autumn and winter, the monkeys primarily used intermediate elevations, with autumn elevations slightly lower than those used in winter (Figure 2). This seasonal pattern was highly consistent across the three annual cycles (Figure 3). Significant seasonal differences in altitude use were detected in all years examined. During 2008–2009, altitude use differed significantly among all four seasons (F_3,3242_ = 642.4, *p* < 0.001), following the pattern: summer > autumn > winter > spring. During 2011–2012, summer and winter did not differ significantly from each other, but both occurred at significantly higher elevations than autumn, while spring remained the lowest (F_3,1979_ = 31.4, *p* < 0.001). A similar pattern was observed during 2014–2015 (F_3,1218_ = 97.9, *p* < 0.001), with summer and winter occurring at comparable elevations and both exceeding autumn and spring in elevation use. Overall, despite some variation in the relative positions of autumn and winter, the three annual cycles consistently showed that *R. bieti* occupied lower elevations in spring, higher elevations in summer, and intermediate elevations during autumn and winter.

### 3.2. Seasonal Variation in Solar Radiation

**Figure 4:**
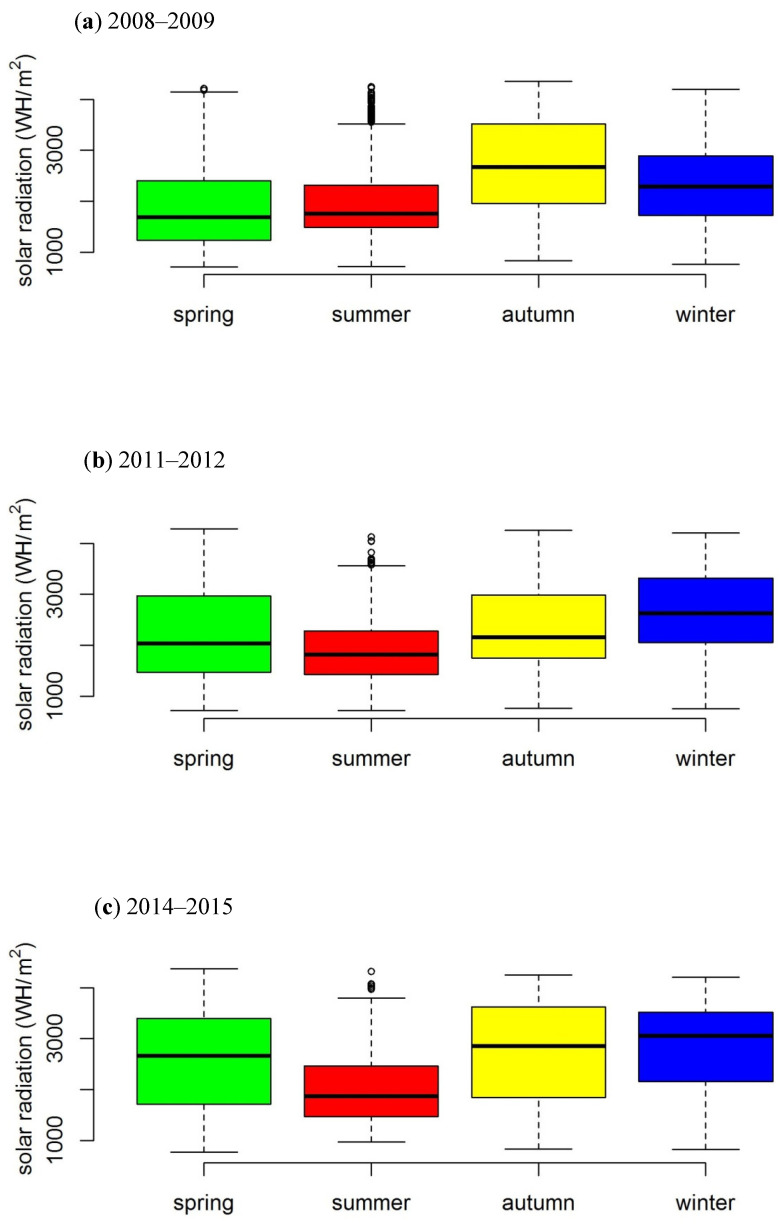
Seasonal variation in winter-solstice solar radiation values at *R. bieti* locations across three annual cycles in the Lasha Mountains.

Solar radiation values associated with *R. bieti* locations differed significantly among seasons in all three annual cycles (ANOVA, all F > 31, all *p* < 0.001; Figure 4). Despite some interannual variation, the overall pattern was highly consistent. Monkeys generally occupied areas receiving higher levels of solar radiation during autumn and winter than during spring and summer. During 2008–2009, solar radiation values at monkey locations were highest in autumn, followed by winter, summer, and spring (F_3,3242_ = 158.4, *p* < 0.001). During 2010–2012, solar radiation was highest in winter, followed by autumn, spring, and summer (F_3,1979_ = 31.4, *p* < 0.001). A similar pattern was observed during 2014–2015, when winter and autumn exhibited the highest solar radiation values and summer the lowest (F_3,1218_ = 40.5, *p* < 0.001). Overall, *R. bieti* consistently used habitats with relatively high solar radiation during the colder seasons (autumn and winter), whereas habitats used during spring and particularly summer were characterized by lower solar radiation values (Figure 4).

### 3.3. Seasonal Variation in High-Quality Food Availability

**Figure 5:**
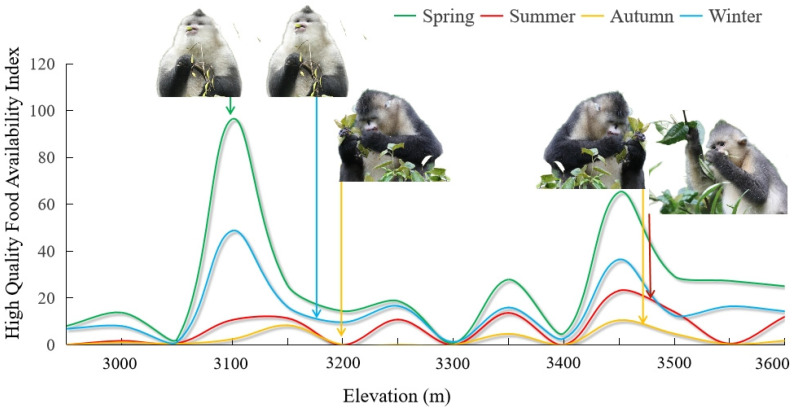
Seasonal variation in high-quality food availability along the elevational gradient in the Lasha Mountains. High-quality food availability is expressed as the Food Availability Index (FAI) for each 50 m elevational belt. Colored arrows indicate the central elevation of monkey distribution in each season.

The distribution of high-quality foods varied markedly across both elevation and season (Figure 5). During winter and spring, high-quality food availability exhibited two peaks, occurring at approximately 3100 m and 3450 m a.s.l. In contrast, during summer and autumn, high-quality food availability was concentrated primarily at higher elevations, with a pronounced peak around 3450 m a.s.l. (Figure 5). The altitudinal distribution of monkey groups showed varying degrees of correspondence with these patterns of food availability. In spring, monkeys were concentrated at approximately 3100 m a.s.l. (Figure 2), coinciding with one of the seasonal peaks in high-quality food availability. During summer, monkeys primarily occupied elevations between 3450 and 3500 m a.s.l., corresponding closely to the elevation range with the greatest availability of high-quality foods. In autumn, monkeys were mainly distributed around 3200 m a.s.l., with a secondary concentration between 3450 and 3500 m a.s.l. Although food availability was relatively low at 3200 m a.s.l., it reached its maximum near 3450 m a.s.l. In winter, monkeys were concentrated between 3150 and 3200 m a.s.l., where the availability of high-quality foods was comparatively low.

### 3.4. Altitude Distribution of Stable Food Abundance

**Figure 6:**
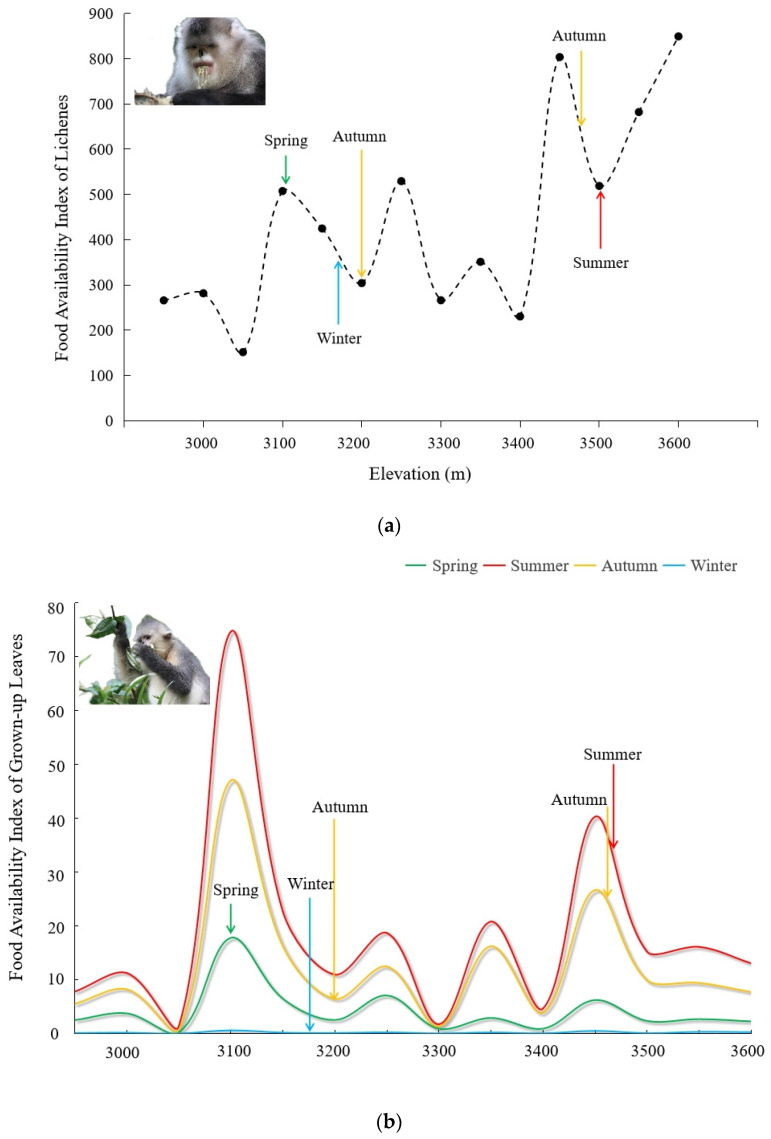
Altitudinal distribution of stable food availability in the Lasha Mountains. (**a**) Lichen availability and (**b**) mature leaf availability along the elevational gradient. Colored arrows indicate the central elevation of monkey distribution in each season (green = spring, red = summer, yellow = autumn, blue = winter).

The availability of lichens, the principal fallback food of *R. bieti*, showed little seasonal variation and remained relatively stable throughout the year (Figure 6a). Across the elevational gradient, lichen abundance was highest between 3100 and 3250 m a.s.l. and at elevations above 3450 m a.s.l. In contrast, the availability of mature leaves varied seasonally (Figure 6b). Nevertheless, their elevational distribution was broadly consistent among seasons, with primary and secondary peaks occurring at approximately 3100 m and 3450 m a.s.l., respectively.

### 3.5. Effects of Solar Radiation and Food Availability on the Seasonal Altitudinal Distribution of R. bieti

**Figure 7:**
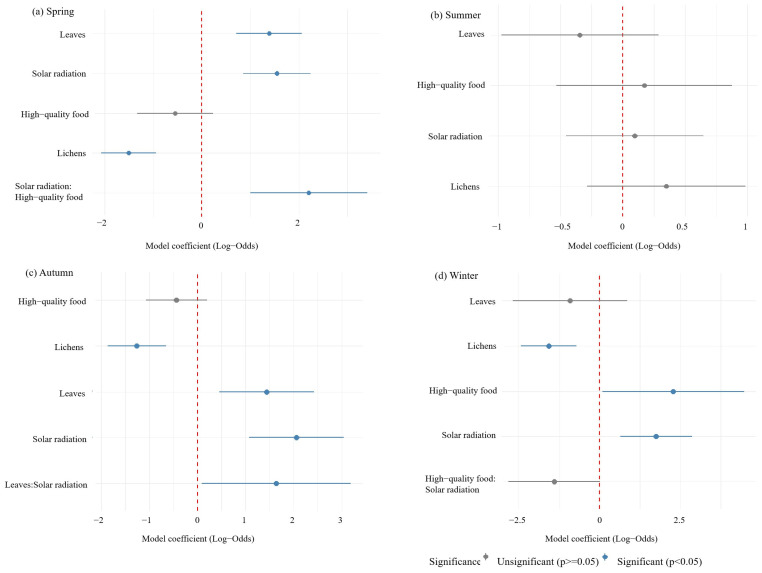
Best-supported GLMs describing the effects of environmental variables on the altitudinal distribution of *R. bieti* in the four seasons. Predictor variables included solar radiation, high-quality food availability, mature leaf availability, lichen availability, and their selected two-way interactions.

The negative binomial regression model incorporating interaction terms provided a strong explanation of the spring altitudinal distribution of *R. bieti*, accounting for approximately 86% of the observed variation (R^2^ = 0.86). Model selection based on AIC identified the model including the interaction between solar radiation and high-quality food availability as the best-supported model (Figure 7a). Both solar radiation and mature leaf availability exhibited significant positive effects on monkey occurrence frequency (Estimate = 1.388, *p* < 0.001). In contrast, the main effect of high-quality food availability was not significant (Estimate = −0.549, *p* = 0.167). However, a highly significant positive interaction was detected between solar radiation and high-quality food availability (Estimate = 2.208, *p* < 0.001). This interaction indicates that the relationship between high-quality food availability and monkey distribution varied with solar radiation levels, with habitats characterized by both high solar radiation and abundant high-quality foods being used more frequently than expected from the additive effects of the two variables alone.

In summer, the best-supported model included four environmental variables as main effects (Figure 7b). However, none of these variables had a statistically significant effect on the altitudinal distribution of *R. bieti*, indicating that variation in monkey distribution during summer was not strongly associated with any of the measured environmental factors.

Environmental variables explained a substantial proportion of the variation in monkey distribution during autumn (R^2^ = 0.497), indicating that the selected environmental variables and their interactions accounted for nearly half of the observed variation in habitat use (Figure 7c). Among the main effects, solar radiation, mature leaf availability, and lichen availability were all significantly associated with monkey distribution, whereas high-quality food availability was not significant. Solar radiation showed a significant positive effect on monkey occurrence frequency (Estimate = 2.061, *p* < 0.001). Mature leaf availability was also positively associated with monkey distribution (Estimate = 1.441, *p* = 0.004). In addition, a significant positive interaction was detected between solar radiation and mature leaf availability (Estimate = 1.641, *p* = 0.037), indicating that the effect of mature leaves on monkey distribution varied with solar radiation levels.

The winter model explained 80.4% of the variation in monkey distribution (R^2^ = 0.804), suggesting a strong association between environmental variables and seasonal habitat use (Figure 7d). Solar radiation had a significant positive effect on monkey distribution (Estimate = 1.747, *p* = 0.002). High-quality food availability, which consisted primarily of buds during winter, was also positively associated with monkey occurrence frequency (Estimate = 2.275, *p* = 0.042). A marginally significant negative interaction was detected between solar radiation and high-quality food availability (Estimate = −1.398, *p* = 0.052), suggesting that the positive effect of one variable tended to weaken as the other increased.

## 4. Discussion

Our results indicate that food resources and solar radiation jointly influence the adaptation of *R. bieti* to high-altitude habitats. Across the three annual cycles, monkeys occupied lower elevations around 3100 m a.s.l. in spring, moved to higher elevations around 3500 m a.s.l. in summer, and shifted to intermediate elevations during autumn and winter. Although lichens constitute an important fallback food for *R. bieti*, lichen availability did not emerge as a significant predictor of seasonal habitat use in the present study. This result may be explained by the relatively high and stable abundance of lichens throughout the study area, which likely reduces their importance as a limiting resource. Consequently, seasonal variation in monkey distribution appears to be more strongly associated with changes in the availability of high-quality foods and solar radiation.

In spring, monkeys were primarily distributed around 3100 m a.s.l. (Figure 1), where the availability of high-quality foods, particularly buds and young leaves, was highest along the elevational gradient. However, the statistical analyses suggest that monkey groups respond differently to different food types. For relatively abundant and readily available foods such as mature leaves, monkeys showed a general positive response. In contrast, the utilization of high-quality foods was strongly associated with solar radiation, as indicated by the significant positive interaction between these two factors. This result suggests that monkey groups tend to exploit nutrient-rich foods preferentially in warm and sunny habitat patches. Such a pattern may reflect an energy-maximization strategy in which monkeys simultaneously increase energy intake through the consumption of high-quality foods while reducing thermoregulatory costs through the use of habitats with favorable thermal conditions. As spring represents a period of recovery from winter and increasing reproductive activity, the combined availability of nutrient-rich foods and solar radiation may be particularly important for meeting the elevated energetic demands of this season.

In summer, monkeys were primarily distributed between 3450 and 3500 m a.s.l., where the availability of high-quality foods was greatest. Interestingly, the habitats occupied by monkeys during summer were characterized by the lowest solar radiation values of the year. Consistent with these observations, the GLM analyses indicated that none of the environmental variables considered in this study had a significant effect on monkey distribution during summer. This result suggests that when food resources are abundant and energetic constraints are relaxed, habitat use is not strongly limited by either food availability or solar radiation. Under such conditions, other factors not considered in this study, such as social dynamics, mating behavior, predation risk, or habitat structure, may play a more important role in shaping ranging patterns.

In autumn, monkeys were distributed primarily around 3200 m a.s.l., with a secondary concentration between 3450 and 3500 m a.s.l. Although high-quality food availability reached its maximum at approximately 3450 m a.s.l., there was relatively little variation in the abundance of high-quality foods across the elevational gradient during autumn. The use of elevations around 3200 m a.s.l., where high-quality food availability was comparatively low, may therefore be related to other habitat characteristics. For example, this elevation supports the highest diversity of food species within the study area (unpublished data), potentially providing a more stable and diverse food supply. The autumn model explained approximately 50% of the variation in monkey distribution, suggesting that environmental constraints begin to emerge during this transitional season. Solar radiation and mature leaf availability both had significant positive effects on monkey distribution, and the positive interaction between these factors indicates that monkeys preferentially used habitats where both thermal conditions and readily available food resources were favorable.

In winter, monkeys were concentrated between 3150 and 3200 m a.s.l., where the availability of high-quality foods was relatively low. Lichen abundance at these elevations was also only slightly above the lowest levels recorded across the study area, whereas solar radiation was relatively high. The winter model revealed significant positive effects of both solar radiation and high-quality food availability, together with a negative interaction between these variables that approached statistical significance. This pattern suggests a trade-off between energy acquisition through foraging and energy conservation through thermoregulation. In habitats with high solar radiation and more favorable thermal conditions, the influence of food availability on habitat use was reduced, whereas in less favorable thermal environments, food resources may become increasingly important. Such a trade-off likely reflects an adaptive strategy that enables monkeys to balance the competing demands of energy intake and heat conservation under the energetic challenges imposed by winter conditions.

Different seasonal altitudinal ranging patterns of *R. bieti* have been reported in previous studies. In Xiaochangdu on the southeastern Tibetan Plateau, which represents the northeastern part of the species’ distribution range, monkeys have been reported to use the highest elevations of their habitat during winter [18]. In contrast, different seasonal ranging patterns have been reported from Baima Snow Mountain, located south of Xiaochangdu and representing the central part of the species’ distribution range. In northern Baima Snow Mountain, monkeys were reported to use the highest elevations in summer, the lowest in spring, and intermediate elevations in autumn and winter [21]; however, other studies reported greater use of higher elevations during winter [22,30] or found no clear seasonal altitudinal stratification [20]. In the Samage Forest of southern Baima Snow Mountain, *R. bieti* was reported to occupy the highest elevations in summer [24], while Li et al. [19] also documented a pattern in which monkeys occupied the lowest elevations in spring, the highest elevations in summer, and intermediate elevations in autumn and winter. Similarly, in Jinsichang, approximately 170 km south of Baima Snow Mountain, monkeys were reported to use the highest elevations in summer, the lowest elevations in spring, and intermediate elevations in autumn and winter [23].

The inconsistencies among these reported altitudinal ranging patterns may partly reflect differences in study sites and research methods, which affect the detection and interpretation of range use. In most earlier studies, monkey locations were determined primarily from indirect evidence such as traces and feces [20,22,23], whereas direct observations of monkey groups were employed in more recent studies [18,19,24]. Indirect sign surveys are affected by the differential persistence and detectability of feces across habitats and seasons, which can bias seasonal altitude estimates. Moreover, sampling effort and temporal coverage varied greatly among studies: the largest dataset comprised 1900 location records across 20 months [24], and only Grueter et al. [24] and Li et al. [19] conducted fieldwork in every month of a full year. Incomplete seasonal coverage and small sample sizes can easily fail to capture the full annual range of altitudinal movements, producing divergent patterns even within the same study area [19,20,21]. Beyond research methods, site-specific geographic, phenological, and climatic conditions also contribute to the variation—for example, differences in regional altitudinal gradients, vegetation types that determine the availability of high-quality food, and the use of stable foods, as discussed in the following paragraph.

Different factors have been proposed to drive the altitudinal ranging patterns of *R. bieti*. Most studies have identified food resources as the primary driver [19,23,24], and several studies point to solar radiation [18] and keeping warm [21,22]. Whereas few studies have attempted to integrate these two factors within a single analytical framework, leaving open the question of whether they act independently, synergistically, or conditionally depending on season. For example, Quan et al. [18] proposed a sunshine hypothesis which posits that solar radiation is the primary driver of the monkeys’ use of higher elevations during winter. However, they did not evaluate the role of food resources in shaping altitudinal distribution. In contrast, Grueter et al. [24] found that lichen, the monkeys’ staple food, explained more variation in monthly use of altitudes than climatic factors, but they did not assess the influence of solar radiation.

Our study addresses this gap by simultaneously evaluating the relative contributions of both food resources and solar radiation across all seasons, using a substantially larger and more spatially continuous dataset than previous efforts—spanning multiple years and covering a nearly 2000 m elevational gradient. Our results confirm that the monkeys indeed spend spring in the lowest habitats and ascend to the highest elevations in summer, consistent with earlier reports [19,21,22,23]. More importantly, we reveal a seasonal partitioning of driver importance: solar radiation emerges as a significant predictor only in winter and spring, whereas high-quality food shows explanatory power in spring and autumn. This suggests that neither food nor solar radiation alone can fully account for the monkeys’ seasonal altitudinal behavior; rather, their relative importance varies with the ecological constraints characteristic of each season. In winter, when thermal stress is most severe, solar radiation becomes the limiting factor—consistent with the sunshine hypothesis of Quan et al. [18]. In spring and autumn, food availability gains importance, supporting the resource-driven model of Grueter et al. [24]. Summer, however, appears to be a period of relaxed environmental constraints, during which neither factor strongly predicts elevational distribution—suggesting that behavioral and social factors may play a greater role during the resource-rich season.

In addition, our study simultaneously considered both food resources and solar radiation as potential drivers of seasonal altitudinal ranging. The results suggest that *R. bieti* exhibits considerable ecological plasticity in responding to seasonal changes in resource availability and climatic conditions. In spring, high-quality foods, including bamboo shoots, buds, and young leaves, first become available at lower elevations (unpublished data). Accordingly, monkeys concentrated in lower-elevation habitats and gradually moved upslope as these resources emerged at higher elevations. During summer, monkeys occupied the highest elevations of their home range, corresponding to the elevational zone where bamboo shoots, buds, and young leaves were most abundant. In autumn, fruits at lower elevations ripened earlier, and monkeys gradually shifted downslope from the highest elevations while tracking the progression of fruit availability across the elevational gradient. These seasonal movements suggest that monkeys closely track the spatiotemporal distribution of high-quality foods throughout much of the year, consistent with previous studies that identified food resources and plant phenology as important drivers of altitudinal ranging in *R. bieti*.

The pattern changes in winter, when temperatures reach their annual minimum and thermoregulatory demands become greatest. During this season, monkeys were more strongly associated with habitats receiving high levels of solar radiation. These habitats are often located on lower slopes or in sheltered valleys, where exposure to solar radiation is high and wind chill is reduced, thereby providing more favorable thermal conditions [22]. Similarly, *R. bieti* has been reported to select sunny slopes as sleeping sites [12]. Long-term observations from the Lasha Mountains further showed that winter habitat use shifted to higher elevations between 2009 and 2015 (Figure 5), coinciding with a decline in mean winter temperatures across the three study periods (7.40 °C, 6.39 °C and 5.78 °C, respectively). Although based on a limited number of years, this pattern is consistent with the hypothesis that winter habitat selection is strongly influenced by thermal conditions. Lichens, the principal fallback food of *R. bieti*, may facilitate this winter strategy. Lichens are consumed throughout the year and become particularly important during winter when fruits and young leaves are largely unavailable [31]. In the Lasha Mountains, monkeys spend approximately 80.2% of their annual feeding time consuming lichens and up to 93.2% during winter [25]. Because lichens are abundant and broadly distributed across the landscape, they are unlikely to constrain winter habitat selection. Instead, the stable availability of this fallback resource may allow monkeys to prioritize habitats with favorable thermal conditions. Consequently, selecting areas with high solar radiation appears to be an important behavioral strategy that enables *R. bieti* to cope with the energetic challenges of winter in a high-altitude environment.

Several questions remain open for future investigation. First, our summer models lack significant predictors, suggesting that variables not included here—such as water availability, predation risk, human disturbance, or reproductive stage—may become influential during this season and deserve empirical testing. Second, while we treated lichen biomass as a proxy for food availability, we did not account for spatial variation in its nutritional quality (e.g., protein content, secondary metabolites), which could affect foraging decisions. Finally, given accelerating climate change, future work should examine whether warming temperatures or altered precipitation regimes will shift the seasonality of food availability and thermal stress, potentially disrupting the adaptive altitudinal strategies observed here. Continuous phenological and food monitoring data over many years will be needed in order to more accurately analyze the impact of interannual variations on the movement of monkey populations.

## 5. Conclusions

The seasonal altitudinal ranging patterns of *R. bieti* appear to be shaped by the combined influences of food resources and solar radiation. Solar radiation, which contributes to heat acquisition, emerges as a particularly important driver during the cold season, whereas the relationship between high-quality food availability and solar radiation changes seasonally. Our analyses suggest a shift from a trade-off between food acquisition and thermoregulation in winter to a positive synergy between these factors in spring. This seasonal transition highlights the dynamic behavioral adjustments through which primates respond to changing climatic conditions and resource availability in high-altitude environments. The persistence of *R. bieti* populations may therefore depend on maintaining sufficient behavioral flexibility to cope with seasonal and interannual environmental variation. Nevertheless, although the altitudinal distribution pattern of *R. bieti* was found to be relatively consistent across years, our analyses of food availability were based on a single year of phenological monitoring. Future studies incorporating multi-year phenological data will be important for evaluating the long-term stability of the relationships identified here.

Our findings also have implications for understanding the potential effects of climate change on high-altitude primates. Animals rely on environmental cues to determine the timing and direction of seasonal movements [32], yet such cues may become increasingly unreliable under rapid environmental change [33]. For *R. bieti*, however, the primary threat is more severe: phenological mismatches (i.e., arriving too early or too late). Climate change could lead to extreme weather events that threaten food abundance and availability, which would directly impose negative fitness consequences, including increased mortality or reduced reproductive output [34,35]. Otherwise, because *R. bieti* occupies one of the highest elevational ranges of any primate, it may be particularly sensitive to climatic changes. Previous studies have suggested that monkeys may rely on sunny conditions following snowfall to warm habitats and expose food resources by accelerating snowmelt [18]. Exposure to solar radiation may also provide additional health benefits, such as reducing parasite or skin disease risk [36,37]. Consequently, increases in winter cloud cover, which have been predicted for some mountain regions under climate change scenarios [38], could negatively affect both monkey populations and the broader high-mountain ecosystem. But there may also be some positive effects; for instance, a warmer climate may facilitate monkeys using higher altitudes. Finally, the strong watershed-based distribution pattern exhibited by *R. bieti* highlights the potential value of watershed units as a basis for the long-term conservation and management of wild populations [39]. Together, these results demonstrate that seasonal interactions between food resources and solar radiation play a central role in shaping the altitudinal ranging strategies and behavioral adaptations that allow *R. bieti* to persist in one of the most extreme environments occupied by any primate.

## Data Availability

The original contributions presented in this study are included in the article. Further inquiries can be directed to the corresponding authors.

## Abbreviations

The following abbreviations are used in this manuscript:

GLM	Generalized Linear Models
AIC	Akaike Information Criterion
NDVI	Normalized Difference Vegetation Index
FAI	Food availability index
